# Chronic cerebrospinal venous insufficiency: the end of “The Big Idea”?

**DOI:** 10.1002/brb3.447

**Published:** 2016-02-22

**Authors:** Erwin Stolz

*Brain and Behavior*, 2016; 6(2), doi: 10.1002/brb3.447

In Stolz 2015, the following funding information has been added and this is also reflected in the article online:

“Professor Stolz has received lecture fees from Boehringer Ingelheim, Bracco and Sanofi‐Aventis for lectures on stroke treatment ultrasound contrast agents and neurosonology. In the course of clinical studies he has received support from Schering and Bracco for projects on ultrasound contrast agents and neurosonology. Research projects initiated by him on thrombocyte flow cytometry have received funding from Sanofi‐Aventis.”
